# The effects of newcomers' AI use on organizational identification: the mediating role of newcomer adjustment

**DOI:** 10.3389/fpsyg.2026.1848081

**Published:** 2026-07-09

**Authors:** Na Lu, Baoyi Feng, Renpu Lang, Yingxin Deng, Weipeng Lin, Junjie Ding

**Affiliations:** 1 School of Management, Shandong University, Jinan, China; 2 School of Management, Beijing Institute of Technology, Beijing, China; 3 School of Management, Xi'an Jiaotong University, Xi'an, China

**Keywords:** artificial intelligence (AI), AI use, newcomer adjustment, newcomer socialization, organizational identification

## Abstract

**Purpose:**

While prior research has predominantly emphasized the performance-enhancing benefits of artificial intelligence (AI) use, it has paid limited attention to its potential cost on newcomers' organizational socialization process. Drawing on organizational socialization theory, this study develops a mediation model to examine how newcomers' AI use influences their organizational identification through newcomer adjustment.

**Methods:**

We collected two-wave survey data from 200 newcomers over a one-month interval. Path analysis and bootstrap analyses were conducted to test the proposed theoretical model.

**Results:**

The results showed that newcomers' AI use was negatively related to newcomer adjustment. In turn, newcomer adjustment was positively related to organizational identification. Furthermore, newcomer adjustment mediated the negative relationship between newcomers' AI use and organizational identification.

**Discussion:**

This study extends understanding of AI in the workplace by demonstrating that its consequences are not uniformly beneficial and by revealing how AI use may shape newcomers' early organizational experiences and psychological attachment.

## 1 Introduction

In recent years, the rapid advancement of generative artificial intelligence (Gen AI, hereafter “AI”) has fundamentally reshaped how work is performed (Bankins et al., 2024). As AI tools become increasingly embedded in organizational routines, their use is no longer peripheral but integral to employees' day-to-day task execution (Anthony et al., 2023). This trend is particularly salient for newcomers, who often encounter AI-enabled tools from the very beginning of their organizational entry (Brynjolfsson et al., 2025; Tan et al., 2025). Accordingly, understanding how AI use shapes newcomers' early work experiences has become an important issue for organizational research.

Existing research has paid considerable attention to the implications of workplace AI use, with a predominant focus on employee performance outcomes (Pereira et al., 2023). A growing body of empirical studies shows that AI use can enhance task efficiency, improve decision quality, and augment individual productivity (e.g., Choi et al., 2025; Shao et al., 2025). While these findings have advanced our understanding of the performance benefits of AI, they also reflect an efficiency-centered perspective that emphasizes what AI enables employees to do better. This focus, however, provides a limited view of how AI shapes employees' broader work experiences, particularly in relational and developmental domains. In particular, relatively little is known about how AI use may influence the process through which newcomers become integrated into organizations. This omission is consequential because early socialization experiences play a critical role in shaping employees' long-term attitudes and attachment to the organization (Chow, 2002).

To address this issue, the present study examines the potential consequences of AI use within the context of newcomer organizational socialization. Organizational socialization represents the process through which newcomers acquire the knowledge, relationships, and sense of belonging necessary to function as effective organizational members (Ashford and Black, 1996; Bauer et al., 2007). A central component of this process is the acquisition of tacit knowledge, which is inherently difficult to codify and often transmitted through interpersonal interactions with supervisors and experienced colleagues (Deng et al., 2025; Miller and Jablin, 1991; Nasr et al., 2019). Although AI is highly effective in providing explicit, codified information, it is less suited for facilitating the transfer of tacit knowledge. More importantly, frequent reliance on AI may reduce newcomers' need to seek help from others, thereby decreasing opportunities for interpersonal interaction. Such technological dependence may disrupt the relational foundations of socialization, potentially hindering newcomers' socialization to the organization.

Building on newcomer socialization theory (Bauer et al., 2007), we develop a mediation model that links newcomers' AI use to both proximal and distal socialization outcomes. We focus on newcomer adjustment as a proximal indicator of socialization effectiveness, reflecting the extent to which newcomers have successfully acquired role clarity, social integration, and task mastery (Bauer et al., 2007). Beyond adjustment, a critical distal outcome of the socialization process is organizational identification—that is, the degree to which individuals define themselves in terms of their membership in the organization (Edwards, 2005). We propose that newcomers' AI use may reduce interpersonal interaction, limiting opportunities for tacit knowledge acquisition and thereby impairing newcomer adjustment. Lower levels of adjustment, in turn, weaken newcomers' sense of identification with the organization. In this way, our model captures how AI use may not only affect how newcomers learn to perform their roles, but also how they come to psychologically connect with the organization.

This research contributes to the literature in several ways. First, it shifts attention from performance outcomes to the socialization process, offering a more comprehensive understanding of how AI use shapes employees' early organizational experiences. Second, it provides a more balanced perspective on AI by demonstrating that its effects are not uniformly beneficial but may also carry unintended relational and developmental consequences. Third, by uncovering the mechanism linking AI use to organizational identification through newcomer adjustment, this study sheds light on how technology influences employees' psychological attachment to organizations. Together, these insights underscore the importance of considering both efficiency gains and socialization dynamics in the study of AI in the workplace.

## 2 Theory and hypotheses

### 2.1 Newcomers' AI use and newcomer adjustment

Newcomer adjustment represents the critical process through which individuals transition from “outsiders” to “insiders.” Its essence lies in newcomers' redefinition of their roles, enabling them to achieve social integration, role clarity, and task mastery (Bauer et al., 2007; Chow, 2002). This process relies heavily on access to organizational information and frequent interpersonal interactions. However, within organizational knowledge systems, codifiable and easily transferable explicit knowledge constitutes only a small proportion, whereas the majority consists of tacit knowledge that is highly context-dependent and difficult to formalize or transfer (Barney, 1991; Nonaka, 1994). Such tacit knowledge—including experienced employees' implicit work skills, unarticulated aspects of organizational culture, organizational politics, informal rules, and team interaction norms—can typically only be effectively acquired through close interaction and observation between newcomers and their mentors or colleagues (Miller and Jablin, 1991). Although prior research suggests that AI, as a technological resource, can assist employees in handling standardized tasks (Jia et al., 2024), within the specific context of newcomer socialization, the “non-human” nature of generative AI and the technological dependence they may induce could hinder the adjustment process.

First, frequent AI use may create a “crowding-out effect” on newcomers' interpersonal interactions, thereby disrupting the channels through which tacit knowledge is transmitted. As newcomers become accustomed to seeking immediate and low-cost solutions from AI, their inclination to seek guidance from mentors and communicate with colleagues is likely to decrease significantly. This form of “interpersonal substitution” severs the natural ties through which newcomers build relationships with organizational members, depriving them of opportunities to establish emotional connections and acquire tacit knowledge (e.g., learning implicit organizational values and behavioral norms that are not formally articulated), ultimately hindering deep social integration (Deng et al., 2025; Sedighi et al., 2016).

Second, from the perspective of interpersonal perception, excessive reliance on AI may elicit negative evaluations from team members. Existing research indicates that frequent AI use in the workplace may be perceived by colleagues as a sign of “laziness” or a lack of willingness to engage socially, which in turn reduces their willingness to provide social support and assistance (Li et al., 2024; Zhou et al., 2025). For newcomers who are still in the early stages of organizational socialization, such negative social evaluations from other organizational members may further impede their integration process.

In addition, reliance on standardized content generated by AI may hinder newcomers' understanding of their work roles. The outputs provided by AI are typically based on generalized logic and often fail to capture organization-specific contextual information and complex role expectations (Bertomeu et al., 2025). When newcomers frequently interact with AI to complete tasks, this behavior may stem from an illusion that “AI is more capable than organizational members,” which discourages them from proactively seeking more specialized tacit knowledge (e.g., practical experience, skills, and job-specific cognition) from experienced colleagues or mentors. This, in turn, is detrimental to their ability to understand and fulfill role expectations. Tang et al. (2022) found that AI use may make it more difficult for employees to clarify specific role boundaries and develop a clear understanding of role expectations. Moreover, when employees rely on AI to perform their work, the perceived superiority of AI may lead them to question their own unique value within the workplace, triggering defensive psychological responses when facing the organizational environment, thereby hindering their mastery of role requirements (Yam et al., 2023). Such AI-induced distortions in role cognition and psychological distancing make it difficult for newcomers to accurately understand and adapt to the organizational context. Accordingly, we propose:

Hypothesis 1: Newcomers' AI use is negatively related to newcomer adjustment.

### 2.2 Newcomer adjustment and organizational identification

Organizational identification refers to a deep cognitive process in which individuals psychologically overlap their self-concept with the definition of the organization, thereby developing a sense of belonging and oneness with it (Ashforth and Mael, 1989). According to the organizational socialization literature, newcomer adjustment represents the process through which individual values interact with—and ultimately become integrated into—the organization's vision, mission, and cultural elements (Bogler and Somech, 2002; Cable and Parsons, 2001). Existing empirical studies have demonstrated that effective organizational socialization can significantly enhance employees' organizational identification and affective commitment (Ashforth and Saks, 1996). First, successful work adjustment implies that newcomers have completed the transition from “outsiders” to “insiders,” a process accompanied by the deep internalization of organizational norms and value systems. When newcomers, through the adjustment process, clarify role expectations and acquire the knowledge and skills required for their roles, their motivation to actively accept and integrate into the organizational environment correspondingly increases (Crow and Glascock, 1995). This positive psychological experience encourages newcomers to align their attitudes and behaviors with the organization's value system, to view organizational success as their own, and ultimately to strengthen their identification with the organization. In contrast, if newcomers remain in a prolonged state of maladjustment, they are less likely to develop a clear understanding of organizational goals, which hinders the integration of personal and organizational values and, consequently, the formation of organizational identification.

Second, social integration—an essential dimension of newcomer adjustment—serves as the emotional foundation for the development of organizational identification (Smidts et al., 2001). Chow (2002) noted that the organizational socialization process involves continuous interactions between newcomers and social groups within the organization. When newcomers successfully integrate into teams and establish supportive interpersonal networks, they perceive acceptance and support from the organization. Such positive interpersonal experiences not only enable employees to cognitively recognize themselves as integral members of the organization but also enhance their emotional attachment and affinity toward it. As demonstrated by Ashforth and Saks (1996), well-adjusted employees tend to exhibit higher job satisfaction and lower turnover intentions, which are key manifestations of strong organizational identification. Therefore, as a “proximal outcome” of the socialization process, newcomer adjustment can effectively influence organizational identification as a “distal outcome.” Accordingly, we propose:

Hypothesis 2: Newcomer adjustment is positively related to newcomers' organizational identification.

### 2.3 The mediating role of newcomer adjustment

Building on the above arguments, this study proposes that newcomer adjustment mediates the relationship between AI use and organizational identification. Specifically, the introduction of generative AI tools has altered the way newcomers acquire information after entering organizations. First, newcomers who frequently use AI are less motivated to actively seek interpersonal feedback and build social networks (Miller et al., 2006). This undermines the transmission of tacit knowledge, resulting in a limited understanding of implicit work skills, organizational culture, organizational politics, and norms of interpersonal interaction, thereby hindering newcomers' ability to fully master their roles and achieve deep social integration (Nasr et al., 2019).

Second, impaired newcomer adjustment directly weakens the foundation for the development of organizational identification. When newcomers struggle to adapt to the organizational environment due to a lack of interpersonal support and insufficient tacit knowledge, their psychological inclination to view themselves as “members of the organization” diminishes, making it difficult to develop organizational identification grounded in emotional attachment and value congruence (Mael and Ashforth, 1992). In other words, workplace AI use weakens newcomers' organizational identification because it disrupts the socialization and adjustment process that underpins the formation of identification. Accordingly, we propose:

Hypothesis 3: Newcomer adjustment mediates the relationship between newcomers' AI use and organizational identification. Specifically, newcomers' AI use has an indirect and negative effect on organizational identification through newcomer adjustment.

**Figure 1:**
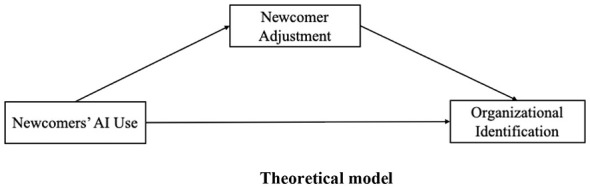
Theoretical model.

The theoretical model of this study is presented in Figure 1.

## 3 Methods

### 3.1 Sample and procedure

The data for this study were collected from newly hired employees at a bank located in North China. During the data collection period, the organization provided access to the complete roster of newly hired employees, yielding a finite population of 387 individuals. Given this fully accessible sampling frame, we adopted a near-census approach by distributing questionnaires to all eligible newcomers, thereby minimizing sampling bias and enhancing the representativeness of the sample. All participants were informed of the study's purpose and assured that their responses would remain confidential and anonymous and would be used solely for academic research purposes.

To mitigate the potential impact of common method bias, we followed the recommendations of Podsakoff et al. (2003) and employed a two-wave time-lagged design, with a 1-month interval between the two waves. In the first wave (T1), data were collected on newcomers' demographic characteristics (e.g., gender, age, and educational level) as well as their use of generative AI. A total of 387 questionnaires were distributed, and 289 valid responses were obtained. 1 month later, in the second wave (T2), questionnaires were administered to respondents who had provided valid responses at T1, primarily measuring newcomer adjustment and organizational identification. A total of 231 questionnaires were returned.

The research team matched responses across the two waves using unique identification codes (i.e., the last four digits of participants' mobile phone numbers). After excluding invalid responses (e.g., those with missing data), a final matched sample of 200 valid responses was obtained, yielding an effective response rate of 51.68%. In the final sample, 104 participants were male (52.00%) and 96 were female (48.00%). The average age was 24.54 years (*SD* = 1.67), with the majority falling between 23 and 25 years old (67.00%). In terms of educational background, 142 participants (71.00%) held a bachelor's degree, while 58 (29.00%) held a postgraduate degree or above. Overall, the sample included newcomers with diverse gender and educational backgrounds, demonstrating good representativeness.

### 3.2 Measures

This study employed well-established scales from prior research to measure the key variables. All items were rated on a five-point Likert scale ranging from 1 (*strongly disagree*) to 5 (*strongly agree*). The translation-back translation procedure was used to translate the measures from English into Mandarin Chinese (Brislin, 1980).

#### 3.2.1 Newcomers' AI use

Newcomers' AI use was measured using the scale developed by Tang et al. (2022), which consists of three items. This scale assessed the frequency and extent to which employees use generative AI in their work. A sample item was “I used artificial intelligence to carry out most of my job functions.” In this study, the Cronbach's α for this scale was 0.98.

#### 3.2.2 Newcomer adjustment

Newcomer adjustment was measured using the scale developed by Haueter et al. (2003), which includes 35 items. This scale was designed to comprehensively assess newcomers' familiarity with and mastery of the organizational environment, team norms, and job tasks. A sample item was “I understand how to act to fit in with what the organization values and believes.” In this study, the Cronbach's α for this scale was 0.99.

#### 3.2.3 Organizational identification

Organizational identification was measured using the scale from Mael and Ashforth (1992), which consists of six items. This scale captured newcomers' sense of belonging and psychological attachment to the organization. A sample item was “When I talk about this organization, I usually say “we” rather than “they”.” In this study, the Cronbach's α for this scale was 0.95.

#### 3.2.4 Control variables

Given that demographic characteristics may influenced newcomers' socialization processes (e.g., Jiang et al., 2021; Li et al., 2011), this study included gender, age, and education as control variables.

### 3.3 Analytical strategy

This study employed SPSS 26.0 and Mplus 8.3 for data analysis. First, we used Mplus 8.3 to estimate the measurement model and test the distinctiveness of the core constructs via confirmatory factor analysis (CFA). Specifically, three key constructs—newcomers' AI use, newcomer adjustment, and organizational identification—were modeled as latent variables, with their respective scale items specified as observed indicators loading onto the corresponding latent factors. Given the relatively large number of items in some scales, we adopted an item parceling strategy in line with the recommendations of Hall et al. (1999) to enhance the stability of model fit indices.

Second, descriptive statistical analyses were performed using SPSS 26.0 to calculate the means and standard deviations of all variables. Pearson correlation analyses were also conducted to examine the relationships among variables, providing preliminary support for the subsequent hypothesis testing.

Finally, hypotheses testing for both the main effects and the mediating effect was conducted using SPSS 26.0 and the PROCESS 3.5 macro developed by Hayes (Model 4). Path analysis and the bootstrap method (with 5,000 resamples and a 95% confidence interval) were employed to test the proposed relationships.

## 4 Results

### 4.1 Confirmatory factor analysis

To examine the discriminant validity of the focal constructs, we specified a three-factor measurement model in which newcomers' AI use, newcomer adjustment, and organizational identification were modeled as distinct latent variables. This baseline model was compared with a series of alternative models, including three two-factor models (in which two constructs were combined) and a one-factor model (in which all items loaded onto a single factor). As shown in Table 1, the three-factor model demonstrated a good fit to the data, meeting commonly accepted criteria (χ^2^ = 42.82, *df* = 24, CFI = 0.99, TLI = 0.99, RMSEA = 0.06, SRMR = 0.04). In contrast, all alternative nested models exhibited significantly poorer fit compared to the three-factor baseline model (Δχ^2^s ≥ 540.87, Δ*df* s ≥ 2). These results indicated that the three core variables in this study possessed satisfactory discriminant validity.

**Table 1:** Confirmatory factor analysis results.

Model	χ^2^	*df*	Δχ^2^(Δ *df*)	RMSEA	SRMR	CFI	TLI
NAIU; NA; OI	42.82	24	–	0.06	0.04	0.99	0.99
NAIU + NA; OI	846.84	26	804.02^***^(2)	0.40	0.23	0.66	0.53
NAIU + OI; NA	628.33	26	585.51^***^(2)	0.34	0.23	0.75	0.66
OI + NA; NAIU	583.69	26	540.87^***^(2)	0.33	0.18	0.77	0.68
NAIU + NA + OI	1,419.36	27	1,376.54^***^(3)	0.51	0.30	0.42	0.23

*N* = 200. NAIU = newcomers' AI use, NA = newcomer adjustment, OI = organizational identification. ^***^ *p* < 0.001.

### 4.2 Descriptive statistics and correlation analysis

The means, standard deviations, and correlation coefficients of all variables are presented in Table 2. The results indicated that newcomers' AI use was significantly negatively correlated with newcomer adjustment (*r* = −0.18, *p* < 0.05), while newcomer adjustment was significantly positively correlated with organizational identification (*r* = 0.30, *p* < 0.01). The directions of these relationships were consistent with theoretical expectations, providing preliminary empirical support for the subsequent hypotheses testing.

**Table 2:** Means, standard deviations, and correlations for variables.

Variable	1	2	3	4	5	6
1.Gender						
2.Age	0.06					
3.Education	0.09	0.76^**^				
4.Newcomers' AI use	−0.19^**^	0.13	0.11			
5.Newcomer adjustment	0.01	−0.08	−0.14^*^	−0.18^*^		
6.Organizational identification	−0.03	−0.02	−0.04	−0.07	0.30^**^	
Mean	1.48	24.54	2.29	2.31	4.13	3.59
*SD*	0.50	1.67	0.46	1.35	0.72	1.04

*N* = 200. Gender was coded “1” for “male” and “2” for “female.” Education was coded “1” for “associate degree or below,” “2” for “bachelor's degree,” and “3” for “master degree or above.” ^*^ *p* < 0.05, ^**^ *p* < 0.01 (two-tailed).

### 4.3 Hypotheses testing

This study employed path analysis to examine the hypothesized relationships, while controlling for gender, age, and education. The results (see Table 3) indicated that newcomers' AI use had a negative effect on newcomer adjustment (*b* = −0.09, *SE* = 0.04, *p* < 0.05), suggesting that higher frequency of AI use among newcomers was associated with lower levels of newcomer adjustment. Thus, Hypothesis 1 was supported. In addition, newcomer adjustment had a positive effect on organizational identification (*b* = 0.43, *SE* = 0.10, *p* < 0.01), indicating that newcomer adjustment could facilitate higher levels of organizational identification. Therefore, Hypothesis 2 was supported. To further test the mediating effect, we conducted bootstrap analyses with 5,000 resamples. The results showed that the indirect effect of newcomers' AI use on organizational identification via newcomer adjustment was negative and statistically significant [indirect effect = −0.04, 95% CI (−0.096, −0.002)], supporting Hypothesis 3.

**Table 3:** Path analysis results.

Variables	Newcomer adjustment	Organizational identification
**Constant**	4.23^**^ (0.89)	1.98 (1.33)
**Control variables**		
Gender	−0.01 (0.10)	−0.08 (0.15)
Age	0.03 (0.05)	−0.00 (0.07)
Education	−0.28 (0.17)	0.03 (0.24)
**Predictors**		
Newcomers' AI use	−0.09^*^ (0.04)	−0.02 (0.06)
Newcomer adjustment		0.43^**^ (0.10)
*R^2^*	0.05	0.09

*N* = 200. Unstandardized coefficients are presented. Standard errors are reported in parentheses. ^*^ *p* < 0.05, ^**^ *p* < 0.01.

To provide a more comprehensive view of the effect decomposition, Table 4 presents the direct, indirect, and total effects of newcomers' AI use on organizational identification. The direct effect was negative but not statistically significant (*b* = −0.02, *SE* = 0.06), whereas the indirect effect was significant. The total effect was negative but not statistically significant [*b* = −0.06, *SE* = 0.06, 95% CI (−0.178, 0.062)]. These findings suggested that newcomers' AI use did not exert a significant direct influence on organizational identification; rather, its negative impact operated primarily through reduced newcomer adjustment. This pattern underscored the role of newcomer adjustment as a key explanatory mechanism linking newcomers' AI use to organizational identification.

**Table 4:** Direct, indirect, and total effects on organizational identification.

Effects	*b*	SE	95% CI
Direct effect	−0.02	0.06	—
Indirect effect (via newcomer adjustment)	−0.04^*^	—	[−0.096, −0.002]
Total effect	−0.06	0.06	[−0.178, 0.062]

*N* = 200. Unstandardized coefficients *(b)* are reported. ^*^ *p* < 0.05.

## 5 Discussion

Drawing on newcomer organizational socialization theory, this study develops a mediation model to examine how newcomers' AI use influences organizational identification through newcomer adjustment. The empirical results indicated that newcomers' AI use was negatively related to newcomer adjustment, newcomer adjustment in turn positively influenced organizational identification, and newcomer adjustment mediated the relationship between AI use and organizational identification.

### 5.1 Theoretical implications

First, this study extends the scope of research on the consequences of workplace AI use by addressing the underexplored technology-socialization interaction perspective. Prior research has predominantly focused on the positive effects of AI use on job performance, decision-making efficiency, or creativity (Anthony et al., 2023; Jia et al., 2024; Lee and Chung, 2024; Sun et al., 2025). Moving beyond the productivity-enhancement perspective, this study adopts an organizational socialization lens and demonstrates that frequent use of AI tools may generate “technological isolation,” thereby threatening newcomers' adjustment process—that is, their transition from “outsiders” to “insiders.” This finding provides new empirical evidence for understanding the complex effects of human-AI interaction in the field of organizational behavior.

Second, this study uncovers the potential negative effects of workplace AI use and validates the “crowding-out effect” of technology on interpersonal interaction. According to organizational socialization theory, the acquisition of tacit knowledge and the formation of emotional bonds rely heavily on interpersonal interaction (Miller et al., 2006). The finding that AI use negatively predicts newcomer adjustment suggests that, although AI efficiently solves standardized problems (i.e., explicit knowledge), its convenience may reduce newcomers' initiative to seek help from mentors or colleagues, thereby disrupting critical channels for building interpersonal trust and understanding organizational culture and informal norms. This challenges a purely technology-deterministic view and highlights the irreplaceable role of human interaction in the socialization process.

Third, this study opens the black box of how newcomers' AI use affects organizational identification by establishing the mediating mechanism of newcomer adjustment. By constructing the mediation model of AI use–organizational identification, this study clarifies the internal process through which technological behaviors translate into psychological outcomes. The results demonstrate that newcomer adjustment, as a proximal outcome, serves as a crucial bridge linking AI use to distal attitudinal variables such as organizational identification. This finding enriches research on the antecedents of organizational identification and suggests that, in the digital era, the formation of organizational identification is shaped not only by organizational context but also by employees' AI use behaviors.

### 5.2 Practical implications

The findings of this study offer several insights for organizational management in the context of digital transformation. First, managers could be aware of the technology-dependence trap during the onboarding stage. Although introducing AI tools (e.g., intelligent Q&A bots or generative AI assistants) can reduce training costs and improve information retrieval efficiency, organizations could recognize that this may come at the expense of newcomers' social integration. Managers could clearly define AI tools as assistive rather than allowing them to fully replace traditional mentoring or onboarding guidance systems.

Second, organizations could design a hybrid onboarding system that balances “high-tech” with “high-touch” elements. For explicit knowledge (e.g., procedural rules, coding standards), newcomers can leverage AI tools for self-learning to improve efficiency. However, for tacit knowledge (e.g., informal work skills, organizational culture, unwritten rules), face-to-face interactions must be preserved or even reinforced. For instance, organizations can design onboarding tasks that require interpersonal collaboration to complete, or establish minimum in-person meeting frequencies between mentors and newcomers to counteract the interpersonal disengagement caused by AI reliance.

Finally, organizations could guide newcomers in developing a correct perspective on AI tool use. Onboarding programs could emphasize that AI is a task-support tool rather than a substitute for relationship-building. While using AI to solve technical problems, newcomers could be encouraged to actively participate in informal team interactions, leveraging interpersonal networks to gain emotional support and social learning. This approach can accelerate organizational socialization and strengthen newcomers' sense of belonging and identification with the organization.

### 5.3 Limitations and future research directions

We acknowledge that there are several limitations in this research. First, the data were collected from a single organization—a bank in North China—which may raise concerns regarding the generalizability of the findings. Although this research design helps enhance internal validity by controlling for organizational-level heterogeneity (e.g., differences in HR practices, technological infrastructure, and organizational culture), it may limit the extent to which the findings can be directly generalized to other contexts. Future research is therefore encouraged to replicate and extend the present model in diverse organizational settings.

Second, although a two-wave survey design was employed to reduce common method bias, the study remains correlational in nature, making it difficult to fully rule out reverse causality (e.g., employees with poorer adaptation may be more likely to rely on AI). Future studies could adopt longitudinal or experimental designs to more precisely capture the formation of AI usage habits and their dynamic causal effects on socialization outcomes.

Third, this study focused primarily on the main effects and mediating mechanism, leaving potential moderators unexamined. For instance, could newcomers' AI literacy, proactive personality, or team inclusiveness mitigate the negative effect of AI use on adaptation? Future research could introduce moderating variables to investigate under what conditions AI's adverse effects can be suppressed and its positive effects amplified, offering more nuanced management guidance for organizations.

## Data Availability

The raw data supporting the conclusions of this article will be made available by the authors, without undue reservation.
